# Differential Entropy-Based Fault-Detection Mechanism for Power-Constrained Networked Control Systems

**DOI:** 10.3390/e26030259

**Published:** 2024-03-14

**Authors:** Alejandro J. Rojas

**Affiliations:** Departamento de Ingeniería Eléctrica, Universidad de Concepción, Concepción 4070409, Chile; arojasn@udec.cl

**Keywords:** networked control systems, AWGN channel, power constraint, differential entropy, fault detection, fault identification

## Abstract

In this work, we consider the design of power-constrained networked control systems (NCSs) and a differential entropy-based fault-detection mechanism. For the NCS design of the control loop, we consider faults in the plant gain and unstable plant pole locations, either due to natural causes or malicious intent. Since the power-constrained approach utilized in the NCS design is a stationary approach, we then discuss the finite-time approximation of the power constraints for the relevant control loop signals. The network under study is formed by two additive white Gaussian noise (AWGN) channels located on the direct and feedback paths of the closed control loop. The finite-time approximation of the controller output signal allows us to estimate its differential entropy, which is used in our proposed fault-detection mechanism. After fault detection, we propose a fault-identification mechanism that is capable of correctly discriminating faults. Finally, we discuss the extension of the contributions developed here to future research directions, such as fault recovery and control resilience.

## 1. Introduction

Control theory developed mainly after World War II. The ideas proposed in this research area are typically related to proportional-integral-differential (PID) control design, state feedback optimal control, optimal observers, and model predictive control (MPC); see [1]. At the turn of the twenty-first century and soon thereafter, the next step was taken under the networked control system (NCS) paradigm, which has evolved into multiagent consensus control [2], cybersecurity [3,4], and data-driven control [5]. Since then, control theorists and control practitioners have remained highly active in this research area, such as by combining control and information theories [6,7] or linear optimal control and communication theory; examples include [8,9,10]. The last few years have also seen an increase in event-triggered NCS solutions [11,12,13], that is, asynchronous control closed-loop solutions, which aim to increase the efficiency of specifically limited communication resources while achieving a set of given objectives (stability, performance, robustness, or a combination of these). These and other NCS results are the foundation of better control.

**Figure 1:**
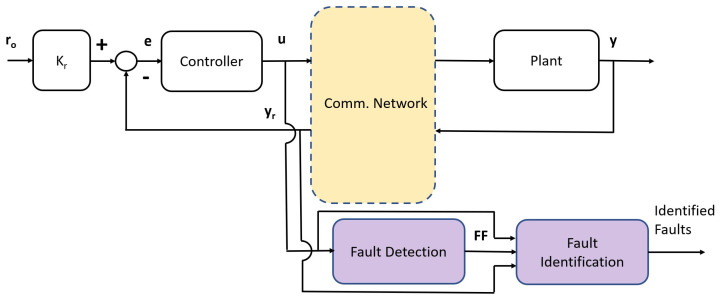
NCS SISO feedback loop with residual generator and fault-detection stages.

An approach to NCS introduced early on by [9] imposes a power constraint, P, on the channel input power and then characterizes the channel model by its channel signal-to-noise ratio (SNR). The proposed SNR approach is then used to study the design constraints on closed-loop stability, especially for cases in which the controlled plant model under analysis is unstable. The SNR limitations presented in [9], which are fundamental in nature, deal with unstable single-input–single-output (SISO) LTI plant models, characterizing the initial bound on the channel SNR required to achieve feedback-loop stability for a single-channel model in the closed loop. A mean square analysis to address the probabilistic nature of the communication network was also used, in a different context of synchronization, in [14]. An NCS extension of the setup proposed in [9] is presented in Figure 1; in this paper, we consider a memoryless additive white Gaussian noise (AWGN) communication channel for the communication network, which operates simultaneously over two paths: the direct path between the controller and the plant and the feedback path between the plant and the controller.

A vast amount of literature also exists on the topic of fault detection and diagnostics, including many published books [15,16,17,18,19] and review articles [20,21]. A fault occurs when there is an anomalous behavior, either by chance or maliciously induced, in a physical plant; it is then important to detect, identify, and if possible recover from this fault. There are different formulations for the problems of fault detection and fault identification for linear time-invariant (LTI) models, which can be roughly categorized as approximate (such as synthesizing fault-detection filters subject to noise) and exact formulations (such as the null-space method).

The variability inherent in NCSs due to the inclusion of stochastic processes in the communication of relevant closed-loop signals might also be caused by variations in the plant model parameters. These parameter variations can be interpreted as faults; thus, a fault mechanism is needed that can detect these changes, identify them, and, through the residuals (see Figure 1), identify the new faulty parameter values to then adapt the controller’s design to achieve fully fault-tolerant closed loop control. A two-part survey on fault diagnosis and fault-tolerant techniques in control can be found in [22,23]. In contrast, the author of [24] offers a complete survey on the topics of fault detection and fault-tolerant control for NCSs. Another survey on fault diagnosis for NCSs can be found in [25], which aims to reduce performance degradation due to communication features. In [26], a fault-detection filter subject to limited transmission through a network with time-varying latency and fading was successfully designed. A Bayesian approach was used instead in [27] for NCS fault diagnosis in an irrigation canal application, while in [28] the authors used a Markov jumping linear system (MJLS) approach for the design of a residual generator. NCS robust fault-tolerant control is also an alternative, which was subsequently considered, for example, in [29]; faults were modeled as MJLSs with incomplete transition probabilities, and LMI-based sufficient conditions were then used to ensure stability. Task allocation in a multiagent setting was presented in [30] to ensure fault tolerance through cooperation between healthy and faulty agents instead of focusing on recovering nominal performance; see also [31]. Finally, a nonlinear MPC solution subject to random network delays and packet dropout was used for fault-tolerant design control in [32].

Our first contribution is the optimal design of the control loop for a first-order unstable plant model, which applies generally due to the nature of the NCS setup. The optimal controller minimizes the sum of the powers for the network input signals u(k) (also the controller output) and y(k) (also the plant model output) in the steady state; see Figure 1. Our second contribution is a fault-detection mechanism based on the estimation of the differential entropy of the controller output signal *u*, as shown in Figure 1. Our third contribution is a fault-identification mechanism for the value of the plant model gain and unstable poles, once a fault has been detected, based on the controller output signal *u* and the received output signal $y_{r}$. We present a simulated example to illustrate these contributions.

This paper is organized as follows. Section 2 presents the general assumptions, introducing the plant and AWGN channel models. Here, we also present the definition of the power of a signal in the steady state. In Section 3, we propose the optimal controller design for the power-constrained NCS control loop. In Section 4, we define the finite-time power estimation and the power constraint-based fault-detection criterion. Section 5 introduces the proposed fault-identification mechanism. In Section 6, we demonstrate the use of both the fault-detection and fault-identification mechanisms based on a simulation example. Finally, in Section 7, we summarize the present work and possible future research avenues.

## 2. Preliminaries

### 2.1. Plant, Channel, and Network Models

We next give the descriptions of the plant, channel, and network models under study.

**Figure 2:**
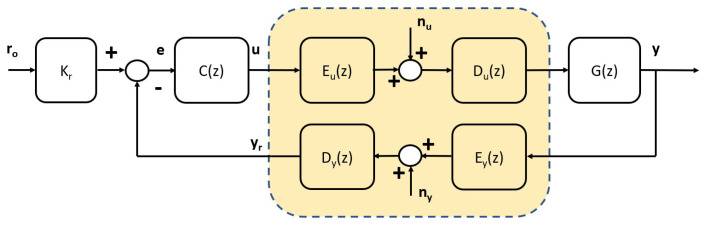
NCS SISO feedback loop with an AWGN network.

**Plant Model**: The plant model, G(z), can be described by a general model given by
  (1) $$G\left(z\right)=G_{s}\left(z\right)\frac{K_{p}}{z-\rho }$$
  where $K_{p}\in R^{+}$, ρ>1, and $G_{s}\left(z\right)$ is a stable, minimum-phase transfer function (i.e., all its poles and zeros are inside the unit circle).
**Channel Models**: The AWGN channel model is described by a channel input power constraint, *P*, and an identically independently distributed additive Gaussian noise process, *n*, with zero mean and variance, $\sigma^{2}$.
**Network Model**: We define the network as two AWGN channel models with an encoder and decoders, one of which is on the direct path between the controller and the plant models and one of which is on the feedback path between the plant and the controller models; see Figure 2. Depending on the channel location, we identify the additive noises as $n_{u}$ and $n_{y}$, both of which are identically independently distributed additive Gaussian noise processes with zero means and variances, $\sigma_{nu}^{2}$ and $\sigma_{ny}^{2}$, respectively. Finally, the powers of the channel input signals are denoted as ${∥u∥}_{Pow}^{2}$ and ${∥y∥}_{Pow}^{2}$.

Furthermore, the setpoint signal, $r_{o}$, in Figure 2 is assumed to be a constant known value.

**Remark** **1.** *The decision to use AWGN channel models to characterize the proposed network model implies that we are considering the channel input constraints and the channel additive noises as the main network features. Other network features, such as transmission delays, quantization, and packet losses, can also be considered, but this would require the use of channel models in addition to the proposed AWGN channel model and would make the characterization of the differential entropy of the control signal, u(k), which is the basis of the proposed fault-detection mechanism, more involved due to the effect of the closed loop.*

### 2.2. Stationary Power of a Linear Time-Invariant System Output Signal

**Definition** **1.** *From* [33]*, we denote the power spectral density of a signal s(k) as $S_{s}\left(e^{j\omega T_{s}}\right)$.*

**Definition** **2.** *The $H_{2}$ norm of a discrete-time linear time-invariant system H is defined as*

(2) $${∥H∥}_{2}=\sqrt{\frac{1}{2\pi }\int_{-\pi }^{\pi }{\left|H\left(e^{j\omega T_{s}}\right)\right|}^{2}d\omega }$$

**Lemma** **1** (Stationary power of an LTI system output signal)**.** *For a discrete-time linear time-invariant system H with a weakly stationary stochastic process n(k) at the input, mean $\mu_{n}$, and spectral density*

(3) $$S_{n}\left(e^{j\omega T_{s}}\right)=\sigma_{n}^{2}$$

*the variance of the noise can be determined. Ref.* [34] *shows that if H is stable (here, it is assumed to be stable if all its poles are inside the unit circle), then*

(4) $$\begin{array}{cc} \mu_{s} & =H\left(1\right)\mu_{n}\\ S_{s}\left(e^{j\omega T_{s}}\right) & =H\left(e^{j\omega T_{s}}\right)H\left(e^{-j\omega T_{s}}\right)S_{n}\left(e^{j\omega T_{s}}\right) \end{array}$$

*and the steady-state power of the output signal is then*

(5) $$\begin{array}{cc} {∥s∥}_{Pow}^{2}:=\lim_{k\to \infty }E\left\{s^{2}\right\} & =H^{2}\left(1\right)\mu_{n}^{2}+\frac{1}{2\pi }\int_{-\pi }^{\pi }S_{s}\left(e^{j\omega T_{s}}\right)d\omega \\ \; & =H^{2}\left(1\right)\mu_{n}^{2}+{∥H∥}_{2}^{2}\sigma_{n}^{2} \end{array}$$

**Proof.**  Since n(k) is a weakly stationary stochastic process and *H* is stable, *s* is also a weakly stationary stochastic process. If we define $s=\mu_{s}+\bar{s}$, where $\bar{s}$ is a weakly stationary stochastic process with zero mean, then we have

$$E\left\{s^{2}\right\}=\mu_{s}^{2}+E\left\{{\bar{s}}^{2}\right\}$$

Since, by its definition, the covariance function of $\bar{s}$ is equal to the covariance function of *s*, and because the power spectral density is the Fourier transform of the covariance function, we have that $S_{s}\left(e^{j\omega T_{s}}\right)=S_{\bar{s}}\left(e^{j\omega T_{s}}\right)$. As we take the limit k→∞, we obtain

$${∥s∥}_{Pow}^{2}=\mu_{s}^{2}+{∥\bar{s}∥}_{Pow}^{2}$$

From [34], we have (4), which, when replaced with the above expression, yields (5), which concludes this proof. □

In the next section, we use the introduced assumptions and Lemma 1 to propose the design of the NCS control loop in Figure 2.

## 3. Networked Control System Design

We start this section with the following lemma, which references a result from [35]; from this lemma, we begin to construct our optimal controller design proposal.

**Lemma** **2** (sum of convex functions)**.** *Let $f_{i},\;i=1,\cdots ,n$ be given convex functions and $\gamma_{i}$ be given positive scalars. Then, the function*

(6) $$F=\gamma_{1}f_{1}+\cdots +\gamma_{n}f_{n}$$

*is also a convex function.*

**Proof.**  See [35]. □

We continue by establishing the working choices for the encoder and decoder blocks in Figure 2. The presence of these blocks is intrinsic to the NCS setup, and it is one of the reasons these types of systems require an extension, not just an application, of classic control theory results.

**Lemma** **3** (Encoder and decoder design)**.** *The encoder and decoder blocks in Figure 2 for the subsequent NCS design are selected as*

(7) $$\begin{array}{cc} E_{u} & =1,\;D_{u}=G_{s}^{-1}\left(z\right),\\ E_{y} & =1,\;D_{y}=1 \end{array}$$

*where $G_{s}\left(z\right)$ is the stable, minimum-phase part of the proposed plant model; see Section 2.1.*

**Remark** **2.** *We observe that, by using Lemma 3 in Figure 2, we can focus entirely on the first-order unstable part of the plant model.*

In the next lemma, we introduce some intermediate results that consider the controller, C(z), to be a proportional controller; that is, $C\left(z\right)=K_{c}$ for $K_{c}\in R$, which will be required for the optimal controller design $K_{c}^{*}$.

**Lemma** **4** (Convexity of closed-loop squared norms)**.** *The following squared $H_{2}$ norms are convex functions of $K_{c}$, the proportional controller:*

(8) $$\begin{array}{cc} {∥T∥}_{2}^{2} & =\frac{K_{c}^{2}K_{p}^{2}}{1-{\left(\rho -K_{c}K_{p}\right)}^{2}}\\ {∥SC∥}_{2}^{2} & =K_{c}^{2}+\frac{K_{c}^{4}K_{p}^{2}}{1-{\left(\rho -K_{c}K_{p}\right)}^{2}}\\ {∥SG∥}_{2}^{2} & =\frac{K_{p}^{2}}{1-{\left(\rho -K_{c}K_{p}\right)}^{2}} \end{array}$$

*where $T=K_{c}K_{p}/\left(z-\rho +K_{c}K_{p}\right)$ is the closed-loop complementary sensitivity and $S=1-T=\left(z-\rho \right)/\left(z-\rho +K_{c}K_{p}\right)$ is the closed-loop sensitivity.*

**Proof.**  We start this proof with the squared $H_{2}$ norm of *T*. For a proportional controller and the simplified plant model, $K_{p}/\left(z-\rho \right)$, the complementary sensitivity is

$$T\left(z\right)=\frac{K_{c}K_{p}}{z-\rho +K_{c}K_{p}}$$

The squared $H_{2}$ norm of the above transfer function is then

$${∥T∥}_{2}^{2}=\frac{K_{c}^{2}K_{p}^{2}}{1-{\left(\rho -K_{c}K_{p}\right)}^{2}}$$

To obtain its critical points, we take the derivative of $K_{c}$ and solve

$$\frac{{\partial ∥T∥}_{2}^{2}}{\partial K_{c}}=\frac{2K_{c}K_{p}^{2}{\left(1-\left(1-K_{c}K_{p}\right)\right)}^{2}-2K_{c}^{2}K_{p}^{3}\left(\rho -K_{c}K_{p}\right)}{{\left(1-{\left(1-K_{c}K_{p}\right)}^{2}\right)}^{2}}=0$$

After grouping the powers of $K_{c}$ in the numerator, we obtain

$$\frac{{\partial ∥T∥}_{2}^{2}}{\partial K_{c}}=\frac{2K_{c}K_{p}^{2}\left(1-\rho^{2}\right)+2K_{c}^{2}K_{p}^{3}\rho }{{\left(1-{\left(1-K_{c}K_{p}\right)}^{2}\right)}^{2}}=0$$

One critical point is $K_{c}=0$, but this solution is outside the region of $K_{c}$ values that ensures closed-loop stability and is thus not considered. The other critical point is $K_{c}^{*}=\left(\rho^{2}-1\right)/\left(K_{p}\rho \right)$. The second derivative is

$${\left.\frac{\partial^{2}{∥T∥}_{2}^{2}}{\partial K_{c}^{2}}\right|}_{K_{c}=K_{c}^{*}}=\frac{\left(2K_{p}\left(1-\rho^{2}\right)+4K_{c}^{*}K_{p}^{3}\rho \right)\left(1-{\left(1-K_{c}^{*}K_{p}\right)}^{2}\right)+0}{{\left(1-{\left(1-K_{c}^{*}K_{p}\right)}^{2}\right)}^{3}}=\frac{2K_{p}^{2}\rho^{2}}{{\left(\rho^{2}-1\right)}^{2}}>0$$

Thus, $K_{c}^{*}$, the only valid critical point, is a minimum, proving that ${∥T∥}_{2}^{2}$ is a convex function. Now, for ${∥SC∥}_{2}^{2}$, we have that

$${∥SC∥}_{2}^{2}={∥\left(1-T\right)K_{c}∥}_{2}^{2}={∥K_{c}-TK_{c}∥}_{2}^{2}=K_{c}^{2}+K_{c}^{2}{∥T∥}_{2}^{2}$$

Thus, since $K_{c}^{2}$ is a convex function of $K_{c}$, we focus on the remaining part, $K_{c}^{2}{∥T∥}_{2}^{2}$:

$$\frac{\partial K_{c}^{2}{∥T∥}_{2}^{2}}{\partial K_{c}}=\frac{-2K_{c}^{5}K_{p}^{4}+6K_{c}^{4}K_{p}^{3}\rho +4K_{c}^{3}K_{p}^{2}\left(1-\rho^{2}\right)}{{\left(1-{\left(\rho -K_{c}K_{p}\right)}^{2}\right)}^{2}}=0$$

The value $K_{c}=0$ is a critical point with a multiplicity of three, but again, it is outside the range of values required for closed-loop stability for $K_{c}$. The other two potential solutions are

$$K_{c1}^{*}=\frac{\rho +\frac{\rho }{2}-\sqrt{\rho^{2}+8}}{K_{p}},\;K_{c2}^{*}=\frac{\rho +\frac{\rho }{2}+\sqrt{\rho^{2}+8}}{K_{p}}$$

but we observe that $K_{c2}^{*}$ is outside the stability region. The second derivative at $K_{c}=K_{c1}$ yields

$${\left.\frac{\partial^{2}K_{c}^{2}{∥T∥}_{2}^{2}}{\partial K_{c}^{2}}\right|}_{K_{c}=K_{c1}^{*}}=\frac{\left(-10{\left(K_{c1}^{*}\right)}^{4}K_{p}^{4}+24{\left(K_{c1}^{*}\right)}^{3}K_{p}^{3}\rho +12{\left(K_{c1}^{*}\right)}^{2}K_{p}^{2}\left(1-\rho^{2}\right)\right)\left(1-{\left(\rho -K_{c1}^{*}K_{p}\right)}^{2}\right)}{{\left(1-{\left(\rho -K_{c1}^{*}K_{p}\right)}^{2}\right)}^{3}}$$

Simplifying, we obtain

$$\begin{array}{cc} \frac{3\rho }{2K_{p}} & >K_{c1}^{*}\\ -2K_{c1}^{*}K_{p}+3\rho & >0\\ -2{\left(K_{c1}^{*}\right)}^{2}K_{p}^{2}+3K_{c1}^{*}K_{p}\rho & >0\\ -2{\left(K_{c1}^{*}\right)}^{2}K_{p}^{2}+3K_{c1}^{*}K_{p}\rho +3\left[\underset{=0}{\underbrace{-{\left(K_{c1}^{*}\right)}^{2}K_{p}^{2}+3K_{c1}^{*}K_{p}\rho +2\left(1-\rho^{2}\right)}}\right] & >0\\ -5{\left(K_{c1}^{*}\right)}^{2}K_{p}^{2}+12K_{c1}^{*}K_{p}\rho +6\left(1-\rho^{2}\right) & >0\\ -10{\left(K_{c1}^{*}\right)}^{2}K_{p}^{2}+24K_{c1}^{*}K_{p}\rho +12\left(1-\rho^{2}\right) & >0 \end{array}$$

Therefore, we determine that the numerator and thus the overall second partial derivative $\partial^{2}K_{c}^{2}{∥T∥}_{2}^{2}/\partial K_{c}^{2}$ at $K_{c}=K_{c1}^{*}$ are positive and that the only critical point value, $K_{c1}^{*}$, is a minimum, which proves that $K_{c}^{2}{∥T∥}_{2}^{2}$ is a convex function of $K_{c}$ in the stability region; through Lemma 2, ${∥SC∥}_{2}^{2}$ is also a convex function of $K_{c}$ in the stability region. Finally, we focus on the term ${∥SG∥}_{2}^{2}$. For the last squared $H_{2}$ norm expression, ${∥SG∥}_{2}^{2}$, we have

$$\frac{{\partial ∥SG∥}_{2}^{2}}{\partial K_{c}}=\frac{-K_{p}^{3}\left(\rho -K_{c}K_{p}\right)}{{\left(1-{\left(\rho -K_{c}K_{p}\right)}^{2}\right)}^{2}}=0$$

The only critical point in this case is given by $K_{c}^{*}=\rho /K_{p}$, which lies inside the $K_{c}$ stability region for the closed loop; when replaced in the second partial derivative, it results in

$${\left.\frac{\partial^{2}{∥SG∥}_{2}^{2}}{\partial K_{c}^{2}}\right|}_{K_{c}=K_{c}^{*}}=2K_{p}^{4}>0$$

Thus, the critical point, $K_{c}^{*}$, is a minimum, and the function ${∥SG∥}_{2}^{2}$ is convex in the $K_{c}$ stability region for the closed loop; this concludes the proof. □

We next use the results just obtained to show the convexity, in terms of the proportional controller, of the power expression for the NCS input signals *u* and *y*; see Figure 2.

**Lemma** **5** (Channel input powers)**.** *For the setup depicted in Figure 2 with $K_{r}=T^{-1}\left(1\right)$, the channel input powers are*

(9) $$\begin{array}{cc} {∥u∥}_{Pow}^{2} & =\frac{{\left(1-\rho \right)}^{2}}{K_{p}^{2}}r_{o}^{2}+{∥T∥}_{2}^{2}\sigma_{nu}^{2}+{∥SC∥}_{2}^{2}\sigma_{ny}^{2}\\ {∥y∥}_{Pow}^{2} & =r_{o}^{2}+{∥SG∥}_{2}^{2}\sigma_{nu}^{2}+{∥T∥}_{2}^{2}\sigma_{ny}^{2} \end{array}$$

*and they are both convex functions of $K_{c}$, the proportional controller.*

We are now ready to use all the previous intermediate results to present the optimal design of the proportional controller, which minimizes the sum of the channel input powers.

**Proof.**  According to Figure 2 and Lemma 3, the signals at the respective channel inputs are

$$\begin{array}{cc} u & =\frac{z-\rho }{K_{p}}r_{o}-T\left(z\right)n_{u}-S\left(z\right)C\left(z\right)n_{y}\\ y & =r_{o}+S\left(z\right)G\left(z\right)n_{u}-T\left(z\right)n_{y} \end{array}$$

We then apply Lemma 1 for z=u and for z=y and obtain the expressions in Equation (9). Finally, since all the squared $H_{2}$ elements of Equation (9) are convex functions of $K_{c}$ as in Lemma 4, together with Lemma 2, it is shown that the channel input powers in Equation (9) are convex functions of $K_{c}$, which concludes this proof. □

**Theorem** **1** (NCS controller design)**.** *The proportional controller, $K_{c}$, is designed so that*

(10) $$K_{c}^{*}=\underset{K_{c}}{argmin}\;{\eta ∥u∥}_{Pow}^{2}+\left(1-\eta \right){∥y∥}_{Pow}^{2}$$

*with 0<η<1, and its optimal value is the unique solution, in the $K_{c}$ stability region $\left[\left(\rho -1\right)/K_{p},\;\left(\rho +1\right)/K_{p}\right]$ for the closed loop, of the following polynomial:*

(11) $$\begin{array}{cc} a_{4} & K_{c}^{4}+a_{3}K_{c}^{3}+a_{2}K_{c}^{2}+a_{1}K_{c}+a_{0}=0\\ \; & \\ a_{4} & =-2K_{p}^{3}\rho \\ a_{3} & =9K_{p}^{2}\rho^{2}\eta \sigma_{ny}^{2}\\ a_{2} & =2K_{p}^{3}\rho \eta \sigma_{nu}^{2}+\left[2K_{p}^{3}\rho \left(1-\eta \right)+\left(8K_{p}\rho -8K_{p}\rho^{3}\right)\eta \right]\sigma_{ny}^{2}\\ a_{1} & =2K_{p}^{2}\left[\left(1-\rho^{2}\right)\eta +K_{p}^{2}\left(1-\eta \right)\right]\sigma_{nu}^{2}+2\left[K_{p}^{2}\left(1-\rho^{2}\right)\left(1-\eta \right)+{\left(1-\rho^{2}\right)}^{2}\eta \right]\sigma_{ny}^{2}\\ a_{0} & =-2K_{p}^{3}\rho \left(1-\eta \right)\sigma_{nu}^{2} \end{array}$$

**Proof.**  From Lemma 5, we have that

$$\begin{array}{c} {\eta ∥u∥}_{Pow}^{2}+\left(1-\eta \right){∥y∥}_{Pow}^{2}=\eta \left[\frac{{\left(1-\rho \right)}^{2}}{K_{p}^{2}}r_{o}^{2}+{∥T∥}_{2}^{2}\sigma_{nu}^{2}+{∥SC∥}_{2}^{2}\sigma_{ny}^{2}\right]+\;\;\;\;\;\;\;\;\;\;\;\;\;\;\;\\ \left(1-\eta \right)\left[r_{o}^{2}+{∥SG∥}_{2}^{2}\sigma_{nu}^{2}+{∥T∥}_{2}^{2}\sigma_{ny}^{2}\right] \end{array}$$

According to Lemma 2, this is a convex function of $K_{c}$, thus characterizing the critical point of the above functional results in obtaining the optimal $K_{c}^{*}$ that minimizes the linear combination of channel input powers. We then take the partial derivative of $K_{c}$, which results in the polynomial $a_{4}K_{c}^{4}+a_{3}K_{c}^{3}+a_{2}K_{c}^{2}+a_{1}K_{c}+a_{0}=0$, with coefficients defined as in (11). Since the proposed functional is convex in $K_{c}$, there is only one critical point in the $K_{c}$ stability region for the closed loop, which concludes this proof. □

**Remark** **3.** If the plant pole ρ (see (1)) is stable, that is, if |ρ|<1, then the minimal channel input powers ${∥u∥}_{Pow}^{2}$ and ${∥y∥}_{Pow}^{2}$ will be zero, and the optimal controller $K_{c}^{*}$ from Theorem 1 will also then be equal to zero, nevertheless resulting in a stable closed loop (although it is technically open if the controller is zero). The fault-detection and fault-identification mechanisms described in the next sections will be applicable as long as a non-zero suboptimal controller is in place for ρ<1 to effectively close the loop.

**Remark** **4.** *Due to the standing assumptions regarding $r_{o}$, the choice of $K_{r}$ in Figure 2, and the relationship between stationary power and signal variance, we have that the NCS controller design proposed in Theorem 1, which minimizes the network input power, can also be interpreted as a minimal-input-entropy controller design.*

The optimal controller design from Theorem 1 results in a stable closed loop, and we now wish to extend its analysis to the case with faults on the two main parameters involved in the optimal controller design, namely, the gain, $K_{p}$, and the plant unstable pole, ρ. We obtain two contributions: a fault-detection mechanism and a fault-identification mechanism. Therefore, we continue by presenting the proposed fault-detection mechanism in the next section.

## 4. Fault-Detection Mechanism

The signal *u* is assumed to be available because it is the result of signal processing through the controller, as shown in Figure 1. On the other hand, the availability of the signal $y_{r}$ requires the assumption of an added sensor at the output of the AWGN channel over the feedback path. Moreover, due to the presence of the channel additive processes $n_{u}$ and $n_{y}$, we cannot consider the instant values of the relevant signals *u* and $y_{r}$, as shown in Figure 1, as representative values. We therefore address this issue by using the average estimates of *u* and $y_{r}$ instead, as shown in the next lemma.

**Lemma** **6** (Finite time estimate)**.** *The averaged signal is obtained as*

(12) $$u_{1}\left(k\right)=\frac{\sum_{k-L+1}^{k}u\left(k\right)}{L}$$

*where L satisfies*

(13) $$\sigma_{u_{1}}^{2}\left(L\right)<\epsilon$$

*for a user-defined tolerance value ϵ.*

**Proof.**  Lemma 5 shows that

$$\mu_{u}=\lim_{k\to \infty }E\left\{u\right\}=\frac{K_{p}}{1-\rho }r_{o}$$

In a stationary state, we then have that $u_{1}$, as defined in (12), will approach $\mu_{u}$ as L→∞ and $\sigma_{u_{1}}^{2}\left(L\right)\to 0$ since $\mu_{u}$ is a constant value, which shows that there will always be a suitable finite value of *L* for any given choice of tolerance ϵ, which concludes this proof. □

**Remark** **5.** *The use of the previous lemma extends in exactly the same way for signal $y_{r}$, for which $y_{r1}$ represents the L average. However, such a signal is only required for the fault-identification mechanism that we propose in the next section.*

**Remark** **6.** *The application of Lemma 6 is based on a Monte Carlo simulation of the NCS-designed control closed loop in steady state with no faults. The selected value of L, through the choice of ϵ, will be a user-selected trade-off between the successful rejection of the noise processes (the larger the L value is, the better) and the responsiveness to the presence of faults (the smaller the L value is, the better).*

We now present our proposed fault-detection mechanism in the following theorem.

**Theorem** **2** (fault-detection mechanism)**.** *Given the setup in Figure 2 for the NCS defined by Lemma 3, with the controller designed as in Theorem 1, the fault flag signal, FF(k), is defined as*

(14) $$FF\left(k\right)=\left\{\begin{array}{cc} 1 & ,\left|\hat{h}\left(u\right)-h\left(u\right)\right|>\delta \\ 0 & ,\left|\hat{h}\left(u\right)-h\left(u\right)\right|\leq \delta \end{array}\right.$$

*where*

(15) $$\hat{h}\left(u\right)=\frac{1}{2}\log_{2}\left[2\pi e\left(u_{2}-u_{1}^{2}\right)\right]$$

*is the estimated differential entropy of the signal u(k), with the time estimate $u_{1}$ defined in Lemma 6 and $u_{2}$ defined as*

(16) $$u_{2}\left(k\right)=\frac{\sum_{k-L+1}^{k}u^{2}\left(k\right)}{L}$$

*Additionally,*

(17) $$h\left(u\right)=\frac{1}{2}\log_{2}\left[2\pi e\left({∥T∥}_{2}^{2}\sigma_{nu}^{2}+{∥SC∥}_{2}^{2}\sigma_{ny}^{2}\right)\right]$$

*is the theoretical differential entropy of the signal u(k) in the steady state when no fault is present. The fault level, δ, is user defined, and it is selected as $2\sigma_{\hat{h}}$, which is twice the standard deviation of the estimated differential entropy of u(k) when no fault is present.*

**Proof.**  From [36] and the fact that the signal u(k) in Figure 2 is a filtered sum of the driving Gaussian processes $n_{u}$ and $n_{y}$, we have

$$h\left(u\right)=\frac{1}{2}\log_{2}\left[2\pi e\sigma_{u}^{2}\right]$$

where $\sigma_{u}^{2}$ is the variance of the signal *u*. From Lemma 5, we have

$$\sigma_{u}^{2}=E\left\{u^{2}\right\}-\mu_{u}^{2}={∥T∥}_{2}^{2}\sigma_{nu}^{2}+{∥SC∥}_{2}^{2}\sigma_{ny}^{2}$$

which results in the proposed expression for h(u) presented in (17). □

**Remark** **7.** *We observe that the selection of δ in Theorem 2 is a compromise between false negative errors (not detecting a fault when one is present) and false positive errors (detecting a fault when one is not present). If the selected δ value is smaller, then more false positive errors will be detected. If the selected δ value is greater, then more false negative errors will be detected.*

**Remark** **8.** *The use of differential entropy for the proposed fault-detection mechanism is motivated by the presence of the AWGN channel and is also a reasonable choice because it introduces a logarithmic scale (base 2 in this case) for the channel input variance, which can otherwise report very large excursions when subjected to faults, as we will observe in the following sections. Moreover, if we select η=1 in Theorem 1 for the NCS controller design, we can then address the minimal h(u) in (17).*

After a fault has been detected by means of Theorem 2, the next step is to estimate its value, that is, to identify it. The next section focuses on this goal.

## 5. Fault-Identification Mechanism

The faults that the control loop might be subject to are involved in the plant model gain, $K_{p}$, or the unstable pole ρ. Additionally, due to the NCS nature of the proposed closed control loop in Figure 2, for the fault-identification mechanism we only stipulate that we have access to the signals *u* and $y_{r}$. That is, we only stipulate access to signals on the controller side of the network (otherwise, transmission through a communication channel would be required); see Figure 1.

As a first step in identifying the detected faults, as described in the previous section, we consider the online estimation of the plant parameters $K_{p}$ and ρ.

**Lemma** **7** (Plant parameter estimation)**.** *From Figure 1, assuming the online availability of signals u(k) and $y_{r}\left(k\right)$ and a selected value of L from Lemma 6, we obtain*

(18) $$\begin{array}{cc} \alpha (k) & =\frac{y_{r1}\left(k\right)}{K_{r}r_{o}}\\ \beta (k) & =\frac{u_{2}\left(k\right)-u_{1}{\left(k\right)}^{2}-{\left(K_{c}^{*}\right)}^{2}\sigma_{ny}^{2}}{\left(\sigma_{nu}^{2}+{\left(K_{c}^{*}\right)}^{2}\sigma_{ny}^{2}\right)\alpha \left(k\right)} \end{array}$$

*and the plant parameter estimates are*

(19) $${\hat{K}}_{p}\left(k\right)=\frac{2\alpha (k)\beta (k)}{K_{c}^{*}\left(\alpha^{2}\left(k\right)+\beta \left(k\right)\right)}\;\hat{\rho }\left(k\right)=1+2\frac{\beta \left(k\right)\left(\alpha (k)-1\right)}{\alpha^{2}\left(k\right)+\beta \left(k\right)}$$

**Proof.**  We first observe that

$$y_{r1}\left(k\right)\approx T\left(1\right)K_{r}r_{o}$$

where $y_{r1}\left(k\right)$ is the *L*-length finite-time estimation of the steady-state value of $y_{r}$. From this, we obtain

$$\alpha \left(k\right)=\frac{y_{r1}\left(k\right)}{K_{r}r_{o}}$$

On the other hand, we have

$$\frac{u_{2}\left(k\right)-u_{1}^{2}\left(k\right)-{\left(K_{c}^{*}\right)}^{2}\sigma_{ny}^{2}}{\sigma_{nu}^{2}+{\left(K_{c}^{*}\right)}^{2}\sigma_{ny}^{2}}\approx {∥T∥}_{2}^{2}$$

and thus

$$\frac{u_{2}\left(k\right)-u_{1}^{2}\left(k\right)-{\left(K_{c}^{*}\right)}^{2}\sigma_{ny}^{2}}{\sigma_{nu}^{2}+{\left(K_{c}^{*}\right)}^{2}\sigma_{ny}^{2}}\approx \frac{\left(K_{c}^{2}\right)K_{p}^{2}}{1-\rho_{c}^{2}}=\alpha \left(k\right)\beta \left(k\right)$$

With these two intermediate results, after algebraic manipulation we obtain the estimate expressions in (19), which concludes this proof. □

We now use Lemma 7, together with the fault-detection mechanism from the previous section, to identify the fault. We provide this result in the next theorem.

**Theorem** **3** (fault-identification mechanism)**.** *The values of the plant fault parameters are identified as*

$$F_{K_{p}}\left(k\right)=\left\{\begin{array}{cc} {\hat{K}}_{p}\left(k\right) & ,\left|K_{p}-{\hat{K}}_{p}\left(k\right)\right|\geq 2\sigma_{K_{p}}\wedge FF=1\\ K_{p} & ,\left|K_{p}-{\hat{K}}_{p}\left(k\right)\right|<2\sigma_{K_{p}}\wedge FF=1\\ K_{p} & ,FF=0 \end{array}\right.$$

*for the plant parameter, $K_{p}$, where $\sigma_{K_{p}}$ is the standard deviation of the plant gain estimation when no fault is present and*

$$F_{\rho }\left(k\right)=\left\{\begin{array}{cc} \hat{\rho }\left(k\right) & ,\left|\rho -\hat{\rho }\left(k\right)\right|\geq 2\sigma_{\rho }\wedge FF=1\\ \rho & ,\left|\rho -\hat{\rho }\left(k\right)\right|<2\sigma_{\rho }\wedge FF=1\\ \rho & ,FF=0 \end{array}\right.$$

*for the plant parameter ρ, where $\sigma_{\rho }$ is the standard deviation of the unstable plant pole estimation when no fault is present.*

**Proof.**  Fault identification is the result of intersecting the estimated plant parameters ${\hat{K}}_{p}$ and $\hat{rho}$ from Lemma 7 with the fault flag signal FF(k) from Theorem 2. Whether the fault is due to $K_{p}$, ρ, or a change in the values of both $K_{p}$ and ρ, the type of fault will be identified as long as the excursion in the value of the faulty parameter exceeds twice the standard deviation of the estimated parameter value when no fault is present. That is, we use the same approach proposed in Theorem 2, but now we validate the fault on either or both plant parameters. □

We have now finalized the theoretical development of this work, and we proceed in the next section to illustrate the proposed contributions through a simulation example summarizing all the previous key points.

## 6. Example

In this section, we develop an example to illustrate the contributions developed in the previous sections. We consider the plant model

(20) $$G\left(z\right)=\frac{2}{z-3}$$

That is, we assume for simplicity here, without loss of generality, that $G_{s}\left(z\right)=1$. The setpoint signal is $r_{o}=0.5$, and the channel additive noise variances are selected as $\sigma_{nu}^{2}=\sigma_{ny}^{2}=0.3$. The NCS proportional controller design from Theorem 1, with an equal weight η=0.5 to equally weight the power contribution of each channel input, results in $K_{c}^{*}=1.2921$. The plant model parameters, and thus the closed control loop, are subject to the following changes for $K_{p}$:

(21) $$\begin{array}{cc} K_{pf1} & =\left\{\begin{array}{cc} -0.3\cdot 10^{-3}k+3.5 & 5001\leq k<6000\\ 1.7 & 6001\leq k<11000\\ 0.3\cdot 10^{-3}k-1.6 & 11001\leq k<12000 \end{array}\right.\\ K_{pf2} & =\left\{\begin{array}{cc} 0.7\cdot 10^{-3}k-18.3 & 29001\leq k<30000\\ 2.7 & 30001\leq k<35000\\ -0.7\cdot 10^{-3}k+27.2 & 35001\leq k<36000 \end{array}\right. \end{array}$$

For ρ,

(22) $$\begin{array}{cc} \rho_{f1} & =\left\{\begin{array}{cc} -1.2\cdot 10^{-3}k+23.4 & 17001\leq k<18000\\ 1.8 & 18001\leq k<23000\\ 1.2\cdot 10^{-3}k-25.8 & 23001\leq k<24000 \end{array}\right.\\ \rho_{f2} & =\left\{\begin{array}{cc} 10^{-3}k-26 & 29001\leq k<30000\\ 4 & 30001\leq k<35000\\ -10^{-3}k+39 & 35001\leq k<36000 \end{array}\right. \end{array}$$

We then propose a first fault on the value of $K_{p}$ starting at k=5001 and lasting until k= 12,000, a second fault due to ρ starting at k= 17,001 and lasting until k= 24,000, and a third and final fault due to a simultaneous change in the values of $K_{p}$ and ρ starting at k= 29,001 and ending at k= 36,000.

**Figure 3:**
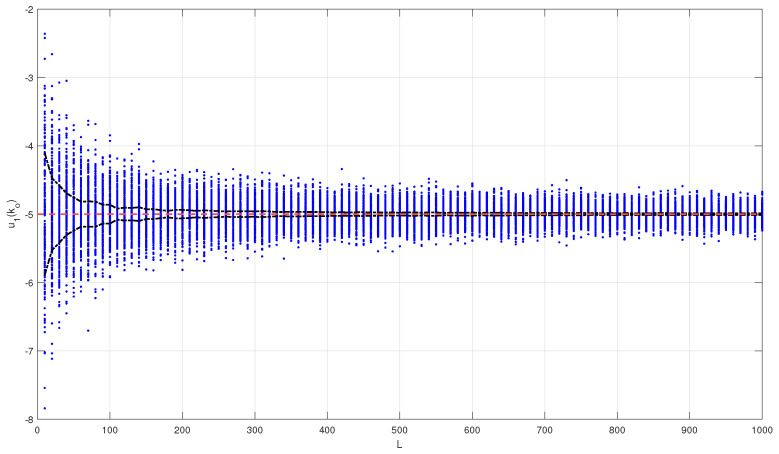
Monte Carlo simulation of $u_{1}\left(k_{o}\right)$ as a function of *L* for $k_{o}=3000$ (blue dots), predicted mean value $\mu_{u}$ (red dashed line), and variance of $u_{1}\left(k_{o}\right)$ as a function of *L* (black dash-dotted line).

The first step is the selection of *L* as a compromise between the rejection of the two noise processes and responsiveness to the faults. In Figure 3, we show a Monte Carlo simulation of two hundred simulations of $u_{1}$ at $k_{o}=3000$ for each value of *L*, in steps of ten. The red dashed line is the steady-state predicted value, $\mu_{u}$, and the black dash-dotted lines are the variances of the two hundred simulations at each value of *L* around the mean value. As predicted, the variance decreases as *L* increases. From Figure 3, the choice of L=300 is considered a good compromise, and it is the value used in the following steps. The proposed selection is compatible with a tolerance value of ϵ=0.04.

**Figure 4:**
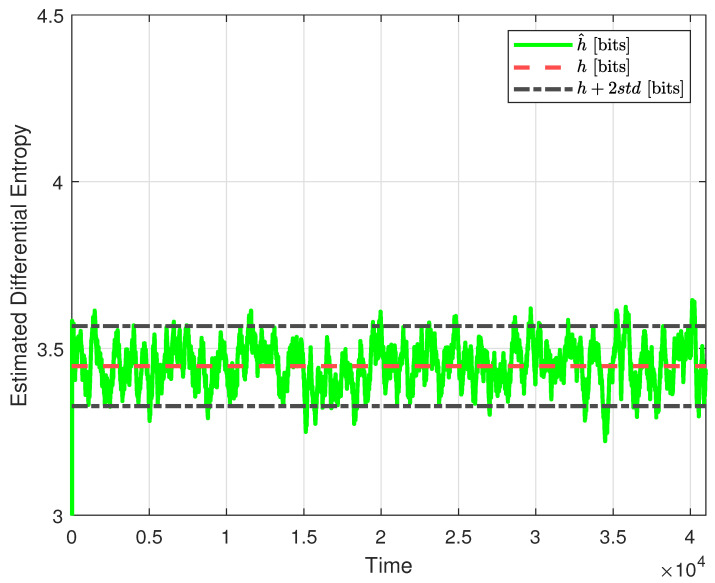
Estimated differential entropy of the signal u(k), when no faults are present, for the selected value of L=300 (green solid line), no-fault theoretical value (red dashed line), and no-fault theoretical value plus two standard deviations (black dash-dotted lines).

As a second step, focusing on Theorem 2, we provide the estimate $\hat{h}\left(u\right)$ (solid green line) and propose from the same figure a choice of δ that is twice the standard deviation of the observed $\hat{h}\left(u\right)$, which in this case amounts to δ=0.12. Therefore, any increase in the estimated differential entropy, $\hat{u}$, of more than 0.12 from the base value, h(u), represented by the red dashed line in Figure 4, is registered as a fault.

**Figure 5:**
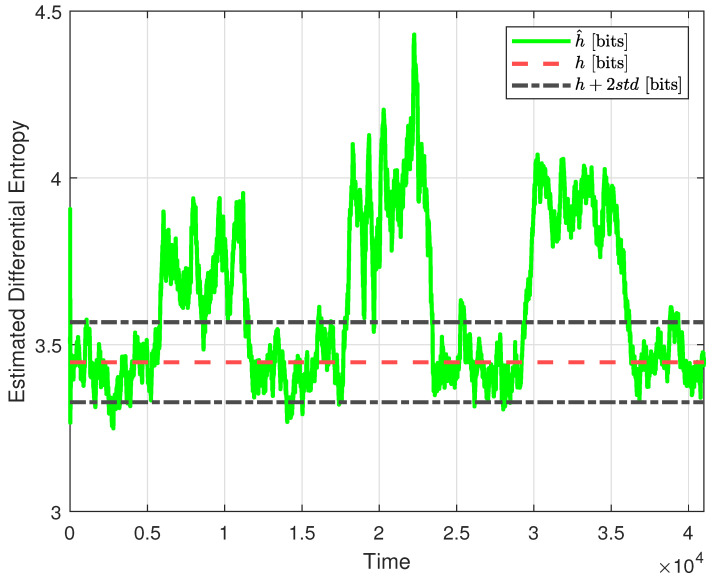
Estimated differential entropy of the signal u(k), when faults in (21) and (22) are present, for the selected value of L=300 (green solid line), no-fault theoretical value (red dashed line), and no-fault theoretical value plus two standard deviations (black dash-dotted lines).

We now test the proposed fault-detection mechanism for the designed NCS closed control loop, with the faults described in (21) and (22). In Figure 5, the three proposed faults can be clearly observed. With the selected value of δ, there will be small instances of false negative errors for $K_{pf1}$ around k=8600 and for $\rho_{f1}$ around k= 19,600. However, no false negative errors are present for simultaneous faults $K_{pf2}$ and $\rho_{f2}$. The choice of δ also triggers some instances of false positive errors around k=1000, k= 16,000, k= 25,000, and k= 39,000. This is the expected trade-off between false positive errors and false negative errors for any fault-detection mechanism.

**Figure 6:**
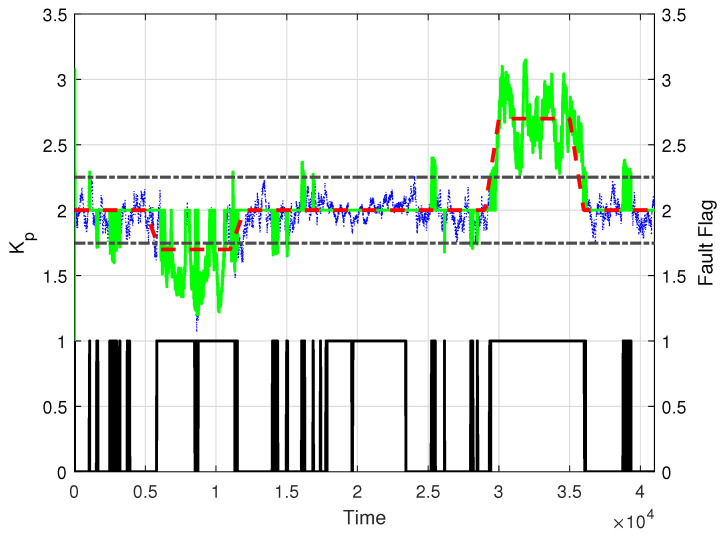
Estimated value of the plant gain parameter $K_{p}$ for the selected value of L=300 (green solid line), true parameter value (red dashed line), standard deviation $\sigma_{k_{p}}$ (black dash-dotted line), and reported fault flag (black solid line at the bottom).

The next step is to couple the fault-detection mechanism of Theorem 2 with the fault-identification mechanism from Theorem 3. The result for $K_{p}$, subject to the proposed faults, is shown in Figure 6. We observe that the inclusion of further discrimination by means of the estimated standard deviation $\sigma_{K_{p}}$, represented by the black dashed line, reduces the effect of false negative errors and false positive errors. Moreover, during the second fault, starting at k= 17,001, which is due only to a change in ρ, the introduction of the $\sigma_{K_{p}}$-based discriminant in Theorem 3 allows the plant model gain to remain at the correct value, $K_{p}=2$.

**Figure 7:**
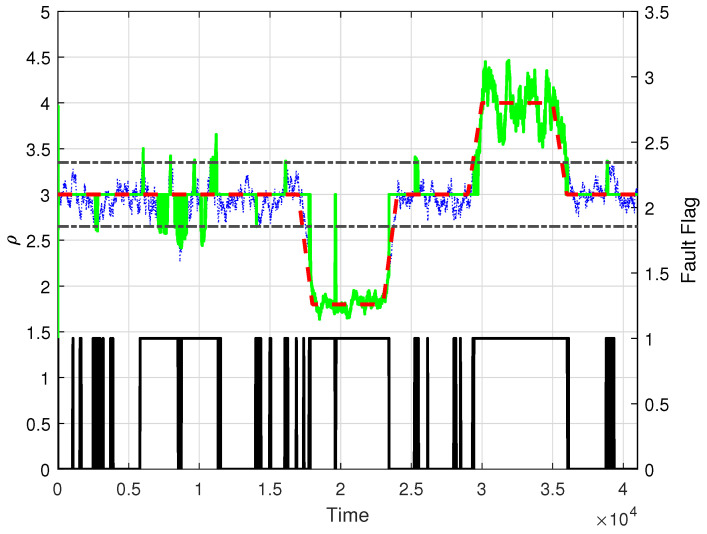
Estimated value of the plant gain parameter, ρ, for the selected value of L=300 (green solid line), true parameter value (red dashed line), standard deviation $\sigma_{\rho }$ (black dash-dotted line), and reported fault flag (black solid line at the bottom).

We conclude the example by reviewing the estimation of the unstable plant pole value, ρ, subject to the faults in Figure 7 (green solid line). As we can see, the introduction of the standard deviation discriminant, $\sigma_{\rho }$, was not as successful as for the plant model gain in avoiding a noisy estimation during the first fault starting at k=5001, even though this first fault is only due to a change in the value of $k_{p}$. Moreover, during the second fault, due only to a change in the value of the unstable plant pole, a false negative error is still present in the proposed identification at approximately k= 20,000. Nevertheless, some instances of false negative errors were suppressed between the first and second faults and at the end of the simulation run.

Finally, we observe that Figure 6 and Figure 7 together demonstrate accurate detection and identification of the faults we introduced into the closed control loop.

## 7. Conclusions

In this work, we propose an optimal NCS design subject to a network of simultaneous power-constrained AWGN channels over direct and feedback paths. The optimal controller design is then the foundation of a differential entropy estimation fault-detection mechanism. The use of differential entropy is justified by the presence of the AWGN channel and is also reasonable since it introduces a logarithmic scale on the channel over the direct-path input variance, which can otherwise result in very large excursions when subjected to faults, as observed in the provided example. The last contribution is a fault-identification mechanism restricted to the signals available on the controller side of the network, namely, *u* and $y_{r}$. A limitation of the proposed fault-detection method is the trade-off imposed by the choice of *L*. The smaller the value of *L* is, the larger the value of δ is because of $u_{1}$ and $u_{2}$, and vice versa. Since the value of δ determines the sensitivity of the fault-detection mechanism, an experienced user must strike the right compromise between these two design parameters. Additionally, as a future research direction, the imposed side restriction signal availability, due to the NCS nature of the closed control loop, can be explored to improve the use of signals on the plant side of the network in the design of a fault-detection/identification mechanism. Finally, once the faults are successfully identified, they should be used for retuning the optimal controller in an adaptive scheme that allows for fault recovery.

## Data Availability

Data is contained within the article.
